# Resolving In Situ Exposure Dynamics in a Chemically Amplified EUV Photoresist Using Table-Top EUV Photoemission Spectroscopy

**DOI:** 10.1021/acsami.5c09589

**Published:** 2025-09-05

**Authors:** Dhirendra P. Singh, Laura Galleni, Faegheh S. Sajjadian, Ivan Pollentier, Fabian Holzmeier, Geoffrey Pourtois, Stefan De Gendt, Michiel J. van Setten, Thierry Conard, John S. Petersen, Paul A. W. van der Heide, Kevin M. Dorney

**Affiliations:** † Imec, Kapeldreef 75, 3001 Leuven, Belgium; ‡ Department of Chemistry, 26657 KU Leuven, Celestijnenlaan 200F, 3001 Leuven, Belgium

**Keywords:** extreme ultraviolet (EUV), lithography, chemically amplified photoresist, secondary electrons, photoemission spectroscopy, electron-induced chemistry

## Abstract

Extreme ultraviolet (EUV) lithography has revolutionized the high-volume manufacturing of nanoscale components. The use of EUV light leads to ionization-driven chemistry in the imaging materials of lithography, the photoresists. The complex interplay of ionization, generation of primary/secondary electrons, and the subsequent chemical mechanisms that lead to image formation in photoresists has been notoriously difficult to study. This is in particular true for the radiochemical transformations occurring during exposure. In this work, we deploy table-top EUV photoemission spectroscopy to observe in situ chemical changes occurring during exposure in a model chemically amplified photoresist and discover a surprising chemical reaction pathway, the EUV-induced breakdown of a perfluoroalkyl substance (PFAS) photoacid generator (PAG). This previously unobserved breakdown of the PFAS PAG, a critical component in the EUV exposure mechanism, manifests as changes in the intensity of the valence band peaks of the EUV photoemission spectrum, which are linked to degradation of the PFAS PAG via an advanced atomistic simulation framework. Our combined experimental and theoretical approach shows that EUV photoemission can simultaneously resolve chemical dynamics and the production of primary and secondary electrons, giving unique insights into the radiochemical transformation of photoresist materials. More generally, our approach also shows that EUV photoemission spectroscopy can provide a unique platform for tracking degradation pathways of PFAS molecules in thin films, owing to the high ionization cross section of fluorine at EUV wavelengths. Our results pave the way for utilizing accessible, table-top EUV spectroscopy systems for observing EUV photoresist chemical dynamics, with the potential for time-resolved measurements of photoemission processes in the future.

## Graphical abstract

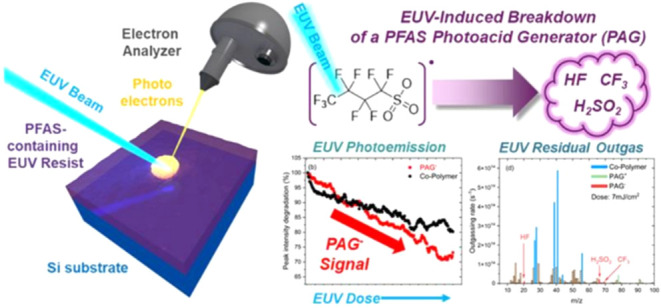

## 1 Introduction

The successful adoption of extreme ultraviolet (EUV) lithography using a wavelength of 13.5 nm (92 eV) into integrated circuits (IC) high volume manufacturing (HVM) at the end of the past decade has pushed the continuation of Moore’s law.^1–6^ The short wavelength of EUV light compared to deep-UV (DUV) light enables a single-print resolution increase of ∼14× (at equivalent numerical aperture and process factors), enabling the production of smaller, denser, and more efficient IC devices.^7^ However, the introduction of ionizing radiation at EUV wavelengths has in turn resulted in a far more complicated exposure mechanism in photoresists in which the aerial optical image is transformed into a chemical image in the resist material. Compared to its DUV predecessor, the exposure mechanism in photoresists is far less understood for EUV exposure.^8–10^ In DUV lithography, a well-understood photochemical process which involves excitation of molecules from ground state to an excited state upon photon absorption, the excited state species are “activated” and can cause additional chemical events that ultimately trigger a solubility change in exposed regions.^10^ In EUV exposure, the absorption of a high-energy photon by valence levels leads to a photoemission process that generates high-energy primary electrons (ca. 55–85 eV). These higher kinetic energy electrons create new pathways for chemical transformations, such as single or multiple ionization, excitation, and electron attachment, followed by ion or neutral dissociation. These processes not only induce new chemical changes in the photoresist but can also lead to the further production of secondary electrons (electrons with kinetic energy <50 eV).^11–14^ Moreover, the chemical products formed from either electron- or photon-initiated chemistries can result in additional byproducts (radicals, ions, etc.) that can cause subsequent chemistries beyond the desired solubility switching reaction. The complicated nature of these intertwined processes has hindered a complete understanding of the EUV exposure mechanism in photoresists, despite the extensive work in this area of the past 20 years.^15–28^

Chemically amplified photoresist (CAR) systems (composed of a polymer base containing varying amounts of photoacid generator (PAG) ion pairs, quenchers, and/or sensitizers/stabilizers, see Figure 1) are the current workhorse of HVM EUV lithography and, as such, much work has been devoted into understanding their chemical transformation upon EUV exposure.^29–32^ Despite the complex radiochemical pathways, the desired outcome in a CAR material is typically a superacid-catalyzed (with the protonated PAG anion being the superacid, often containing a perfluoroalkyl substance (PFAS) molecule) deprotection reaction in which a protecting group (PG) on the polymer chain is removed, thus causing a change in solubility in a developer. This mechanism is initiated by the release of the superacid, which is believed to be started by either low-energy electron attachment/trapping or electron-induced excitation of the PAG cation.^16,33,34^ Given the nonzero EUV absorption cross section of components within a CAR material, it is presumed that primary (and consequently secondary) electrons responsible for initiating this reaction can be generated from nearly any constituent in the resist matrix. Additionally, the direct deprotection reaction initiated by EUV ionization can also occur, as revealed by EUV-induced outgassing experiments.^12,35^ While this general mechanism is supported by theoretical and experimental investigations, the complexity of the exposure mechanism, as well as the chemical diversity of the CAR further complicates spectroscopic tracking of EUV-induced chemical dynamics. Moreover, many experimental techniques do not track chemical changes occurring *during* exposure, thus preventing observation of the in situ EUV-induced chemical dynamics. Finally, a long-standing challenge in unraveling the EUV exposure mechanism is determining the role and yield of photoelectrons in the image formation process as many techniques do not directly observe the photoelectrons generated by 13.5 nm EUV photons.

**Figure 1:**
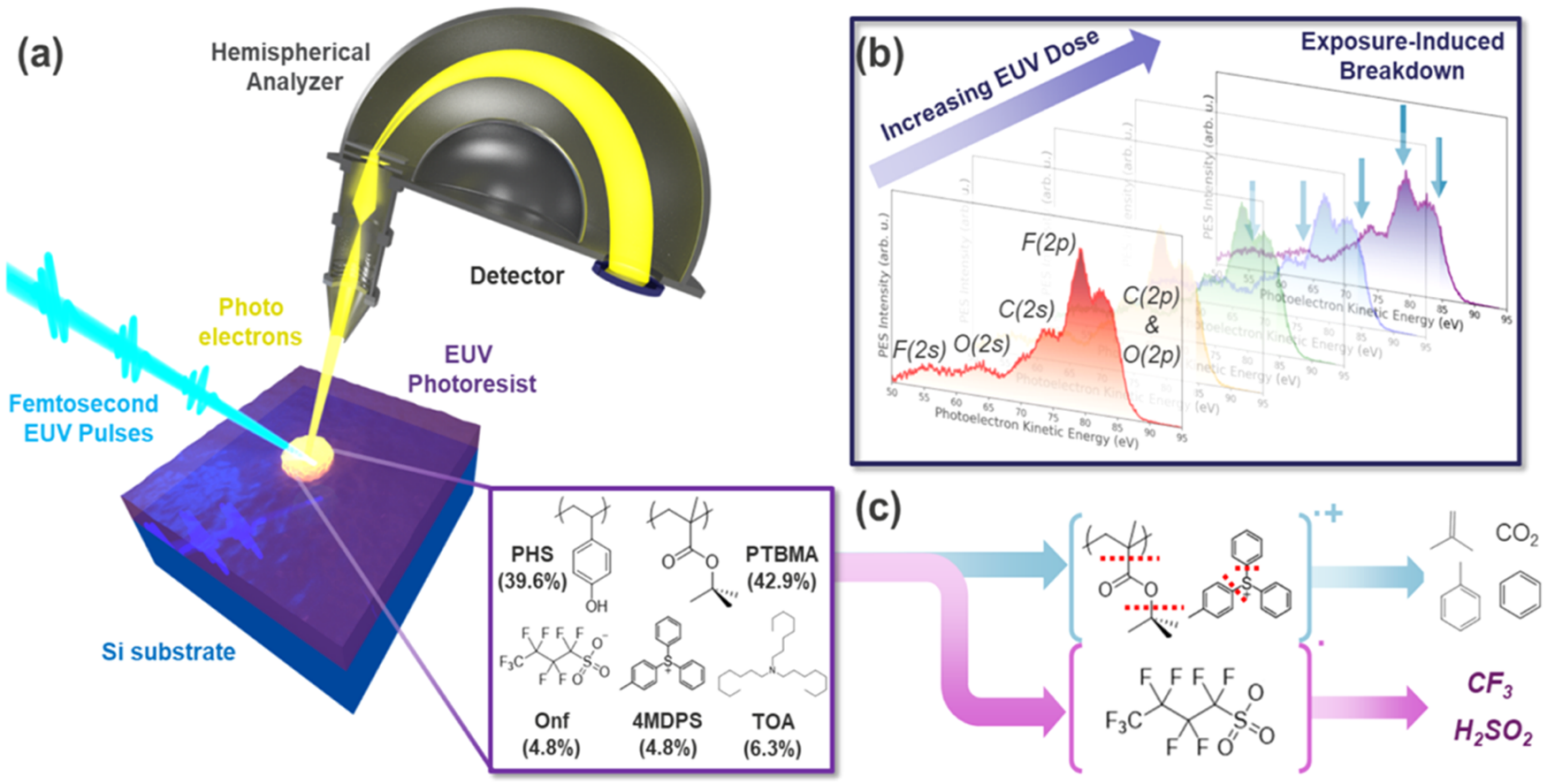
EUV photoemission and exposure-induced breakdown dynamics of the model ESCAP EUV photoresist. (a) Experimental schematic for EUV photoemission spectroscopy of the ESCAP system, which is composed of a femtosecond EUV source based on HHG coupled with a momentum microscope and hemispherical analyzer detection system. The ESCAP system is composed of a polyhydroxystyrene/polytertbutyl methacrylate (PHS/PTBMA) co-polymer with a photoacid generator (PAG) ion pair (nonaflate (ONf), PAG*^–^*, and (4-methylphenyl) diphenyl sulfonium, PAG^+^) and an acid quencher, trioctlyamine, TOA (see text for full composition). (b) EUV photoemission spectra with increasing EUV dose, with peaks assigned by the major atomic orbital contribution. A decrease in the peak intensities is observed as a function of EUV dose, with a prominent decrease coming from the peak with a strong F­(2p) contribution. (c) Photoionization pathways of the ESCAP system after excitation with EUV light, which result in the creation of radical cations in the resist matrix. Typical reaction pathways of PAG^+^ and PTBMA decomposition are indicated in blue, which result in the release of volatile products. The fundamentally new reaction pathway observed in this work, which involves photoionization of the PAG*^–^* and its subsequent breakdown, is indicated by the pink arrows.

Photoemission spectroscopy (PES), in which an incident photon with energy greater than the work function of the material causes the emission of electrons, is a powerful analytical technique (Figure 1a). It can measure the primary and secondary photoelectron spectrum, which provides information on the chemical composition and bonding of the materials. In solids, the inelastic mean free path (IMFP) for photoemitted electrons varies with their kinetic energy, for example, electrons with kinetic energy (*E*_kin_) of 10–150 eV, the IMFP is relatively short (1–2 nm),^36,37^ making it sensitive to surface chemistry. However, in lower-energy electrons, *E*_kin_< 5 eV, the IMFP is relatively larger, thus bringing sensitivity to the bulk properties as well.^36^ Traditionally, X-ray photoelectron spectroscopy (XPS) has been used to quantitatively study the chemistry of thin-film systems. Due to relatively larger IMFP of photoemitted electrons at higher kinetic energy and well-separated core-levels of the atoms, XPS possesses an ideal thickness and chemical sensitivity for chemical analysis of thin-film EUV photoresists.^37,38^ However, the high-energy X-ray photons used in XPS can trigger additional chemical changes in EUV photoresists and the generated photoelectrons are not representative of the photoelectron cascade generated by EUV exposure.^39^ Recently, EUV photoemission using 13.5 nm (92 eV) excitation has been deployed for measuring the generation of primary/secondary photoelectrons in EUV photoresist systems.^15,40,41^ By employing the exposure wavelength of 13.5 nm, key insights relevant to the EUV exposure process have been revealed, such as efficiency of photoelectron production,^42,43^ measurement of the IMFP of primary photoelectrons,^37^ as well as changes in photoelectron production vs exposure dose.^41,44^ Although these studies show that EUV photoemission yields information pertinent to the EUV lithographic exposure process, the results from the EUV photoemission experiments are difficult to interpret. The photoelectrons generated from EUV exposure emerge from valence energy levels that belong to complex molecular orbitals and thus prevent a direct correlation between the photoemission peaks and the chemical nature of the resist. As such, this fundamental limitation has stymied the use of EUV photoemission as a technique for tracking in situ chemical dynamics during exposure. As an additional constraint, EUV photoemission at 13.5 nm has so far been limited to large-scale synchrotron facilities, thus limiting the accessibility of EUV photoemission for studying photoresist exposure dynamics.

In this study, we introduce a combined experimental and theoretical approach that significantly enhances EUV PES as a method for measuring in situ chemical dynamics during EUV exposure in photoresist materials. As illustrated in Figure 1, in situ EUV PES is performed on a variant of the environmentally stable chemically amplified photoresist (ESCAP) platform^45^ (a typical proxy for EUV CAR photoresist systems) using a coherent table-top EUV source based on high-harmonic generation (HHG) coupled with an advanced concentric hemispherical analyzer (CHA). The EUV exposure initiates chemical dynamics, which results in the modulation of the intensity of the valence region peaks in the photoelectron spectrum (Figure 1b). These modulations are interpreted using an advanced *ab initio* simulation toolbox that allows us to correlate changes in the EUV photoemission spectrum with chemical changes occurring in the model CAR system. This synergistic approach enables us to observe and track the degradation of a critical component responsible for the CAR exposure mechanism, the F-based PAG anion (PAG^–^), the conjugate base of perfluorobutanesulfonic acid. The PAG^–^ drives the acid-catalyzed deprotection mechanism and is thus responsible for the solubility switch of image formation in CAR materials. Its degradation by EUV exposure could be one source of chemical stochastic defect generation and ultimately reduction of device yield in HVM processes. The EUV PES results are further supported by XPS experiments on the same resist system that show the removal of F-containing species from the ESCAP film as a function of exposure dose. Potential chemical byproducts of PAG^–^ breakdown are revealed by EUV-induced mass desorption measurements, which indicate release of HF, CF_3_, and H_2_SO_2_ (Figure 1c). Finally, we perform high-sensitivity Fourier transform infrared spectroscopy (FTIR) measurements on the same system that show, in addition to protecting group cleavage, the breakdown of the PAG cation (PAG^+^). Taking together, these results reveal an unexpected reaction pathway in EUV CAR systems, where photoionized PAG^–^ components of the PFAS family significantly contribute to photoelectron yield and may influence the resulting dynamics. Our results not only pave the way for quantitative interpretation of at-wavelength EUV exposure dynamics but also open the door to time-resolved measurements of EUV exposure dynamics with coherent, femtosecond EUV sources.

## 2 Experimental Details

### 2.1 Sample Preparation and Characterization

Photoresist samples were prepared via spin coating of the stock solutions. A modified version of the ESCAP was provided by FUJIFILM. The composition of the modified ESCAP material was as follows: a co-polymer composed of *p*-hydroxystyrene (PHS, 39.6 mol %) and *tert*-butyl methacrylate (PTBMA, 42.9 mol %), (4-methylphenyl) diphenyl sulfonium (PAG^+^) nonaflate (PAG^–^) as the ionic PAG pair (9.6 mol %), and trioctylamine as the quencher (6.3 mol %). To isolate and identify signals arising from the co-polymer and/or the PAG^+^/PAG^–^, samples containing only the co-polymer were also prepared for EUV PES, FTIR, and EUV outgassing measurements. In all photoresist samples, the thickness of the photoresist was determined by fitting reflectance curves from a commercial spectroscopic ellipsometer (RC2, JA Woollam Company). To achieve high-quality fits, blank Si coupons (i.e., with no photoresist) were first measured to determine the thickness of native SiO_2_. The optical constants from these initial fits were then used in the fitting of the ellipsometry data to obtain thickness values of ∼30 nm for the ESCAP material, with a coating uniformity of ±0.5 nm. It is important to note that we do not use metallic underlayers as used in previous measurements of EUV PES on tin-based photoresist materials,^40^ as the additional electrons generated from the metallic underlayer can trigger further reactions in the photoresist that would deviate from the expected behavior in a lithographic scanner environment.

### 2.2 EUV Photoemission Spectroscopy

EUV PES at 13.5 nm excitation is performed using a coherent, femtosecond, table-top EUV system (XUUS 4, KM Laboratories) based on HHG, coupled with an advanced CHA (KREIOS 150, SPECS) employing a hemispherical analyzer for photoelectron detection. The EUV source has been described in detail previously.^46^ Summarizing, the HHG process is performed in helium gas, resulting in a tunable spectrum from ca. 80–120 eV, with a bright, narrowband harmonic at ∼92 eV (13.5 nm, Δλ/λ ∼ 10^–2^). The 92 eV (13.5 nm) harmonic was isolated from the HHG spectral comb using a monochromator that comprised a toroidal focusing mirror, a flat grating mounted in conical diffraction geometry, and a downstream pinhole positioned at the focus of the toroid. The monochromatized 13.5 nm beam was then directed into the KREIOS spectrometer via an additional toroidal focusing mirror with a focal length of 40 cm, resulting in a spot size of approximately 120 × 70 μm^2^ at the sample plane, elongated due to a grazing incidence angle (∼30°). The EUV dose at the sample plane was calculated from the measured spot size at the sample and the measured photon flux from an EUV photodiode inserted into the path of the 13.5 nm beam. The incident EUV photon flux was controlled by inserting thin Zr foils into the beam path. Samples consisting of a modified version of the ESCAP (see Figure 1 for composition), coated on a Si substrate were mounted on a metal plate with a conducting clip to minimize the effects of surface charging and transferred into the analysis chamber (∼5 × 10^–10^ mbar) of the KREIOS instrument for measurements of the photoelectron spectrum (Fermi edge to work function cutoff). The photoelectron spectrum was measured with a pass energy of 50 eV and a slit width of 0.6 mm. The EUV binding energy calibration was performed on the 3d peak of a clean zinc (Zn) sample.

In this setup, the writing speed (EUV exposure dose) can be controlled from ca. 10–100 μJ/s/cm^2^ (dose of ca. 1–10 mJ/cm^2^ for an energy scan of range 5–100 eV). The photoelectron spectrum was measured with an EUV dose of ca. 1–2 mJ/cm^2^. To capture changes in the photoelectron spectra during exposure, the spectra were collected for several hours, leading up to the total EUV dose of approximately ∼212 mJ/cm^2^. Despite this relatively high total dose, the low average power of our HHG-based source ensures minimal localized heating of the sample during exposure; such thermal effects were calculated using Fourier’s law (Supporting Section S6, eq S(1)), and the volatilization of compounds during measurement can be safely neglected.

In all cases, quantitative analysis of the EUV photoelectron spectra was performed by first performing a Tougaard background subtraction to remove contribution from secondary electrons (Supporting Figure S1) and then employing peak deconvolution using the CasaXPS software. The deconvolution of the background-subtracted EUV photoemission spectra was performed using a basis set of mixed Gaussian–Lorentzian functions (90–10) to account for broadening and finite lifetime of the core-hole excited states, respectively. Sets of 7 peaks were used to deconvolute the experimental spectra, which well-reproduced the experimental spectra at all doses (Supporting Figure S2). The peak areas resulting from the deconvolution were then analyzed as a function of in situ exposure dose to quantitatively track EUV-induced dynamics that resulted in changes in the EUV photoelectron spectra.

### 2.3 X-ray Photoelectron Spectroscopy

The XPS measurements were performed on the exposed ESCAP material at doses 0, 20, 60, 100, and 400 mJ/cm^2^ to track the relative loss of F-containing species in the film as a function of exposure dose. The XPS measurements were performed using a QUANTES XPS instrument (Physical Electronics, PHI) employing a monochromatized Al–Kα source (1486.6 eV) with a spot size of ∼100 μm. Samples of the exposed ESCAP were cleaved to 1 × 1 cm^2^ coupons, and XPS spectra of the C­(1s), N­(1s), O­(1s), S­(1s), and F­(1s) were recorded. An electron flood gun was employed for charge neutralization during measurements to prevent charging-induced peak shifts due to the insulating nature of the ESCAP material. The binding energy calibration was performed on the C­(1s) spectrum at 284.8 eV.

### 2.4 Fourier Transform Infrared Spectroscopy

Fourier transform infrared spectroscopy (FTIR) measurements were performed on unexposed and exposed ESCAP samples in transmission geometry using a commercial FTIR spectrometer (iG50, Thermo Fisher Scientific). Samples were placed in a custom sample chamber that consisted of a vacuum chamber containing a goniometer that allows for adjusting the angle of the sample with respect to the incoming IR beam. To increase the signal, samples were inclined at an angle of ∼30° to the beam propagation direction. During the measurement, the sample chamber was evacuated to a pressure of 0.1 mbar to prevent parasitic absorption from H_2_O and CO_2_. FTIR spectra were collected with a resolution of 4 cm^–1^ using 128 scans, which provided sufficient resolution and signal-to-noise to resolve vibrational peaks of the co-polymer and PAG^+^.

### 2.5 EUV-Induced Outgassing

Mass spectrometry analysis of EUV-induced outgassing was performed on the volatile products leaving the resist film during EUV exposure using an established EUV-induced outgassing tool, which has been described previously.^34^ Briefly, the ESCAP material was spin-coated onto 200 mm wafers, and EUV exposure was performed using a spectrally filtered plasma-discharge-based *z*-pinch source (EQ-10, Energetiq). Mass spectra over the range of 0–300 amu were measured during EUV exposure using a Pfeiffer QMG422 quadrupole mass spectrometer, in close proximity (∼5 cm) to the exposed resist. The EUV exposure dose was limited to ∼7 mJ/cm^2^ by raster scanning the EUV beam over the 200 mm wafer. Such low-dose measurements help to track the fragmentation and outgassing of volatile products at lithographically relevant doses. Furthermore, the low dose and large spot size ensure minimal heating of the resist and wafer, ensuring that outgassed products are the result of EUV-induced chemistry and not thermal volatilization (see Supporting Section S6).

The same tool was also used to perform EUV exposures of ESCAP for FTIR measurements. For these exposures, coupon samples (3 × 3 cm^2^) of the coated ESCAP material were fully exposed at doses of 0, 10, 20, 40, 60, 100, 200, and 400 mJ/cm^2^. The exposed samples were then removed from the exposure tool and subsequently measured by spectroscopic ellipsometry for film thickness and FTIR without a postexposure bake.

## 3 Computational Methods

Atomistic model structures of the homo-polymers and of the ESCAP resist were generated using our in-house polymer builder python code based on coarse-grained molecular dynamics. Each structure consists of about ∼1000 atoms.^47^ The simulation box was chosen as a cube of size 2.2 nm (PHS), 2.3 nm (PTBMA) and 2.3 nm (ESCAP) to match the experimental density of 1.0 g/cm^3^.^48^ The model structures were optimized using the Broyden–Fletcher–Goldfarb–Shanno (BFGS) algorithm^49^ using DFT forces calculated using the PBEsol^50,51^ exchange correlation functional and imposing periodic boundary conditions as implemented in the CP2K software package.^52^ The use of the PBEsol functional for the final structural optimization has been benchmarked in ref 47.

To simulate the photoelectron spectra, the density of states (DOS) of each optimized model structure was computed by applying a 0.5 eV Gaussian broadening to the Kohn–Sham energies computed with the HSE06 hybrid functional^53^ and using the standard DZVP basis set^54^ and pseudopotentials^55–57^ provided in the CP2K software package.^52^ The DOS was projected onto individual atoms and angular momentum channels to identify the atomic orbital contributions. Each contribution was then rescaled by the photoionization cross section of the corresponding atomic orbital. The values of the photoionization cross section were obtained for each atomic orbital by a B-spline interpolation of the values tabulated in ref 58 for a photon energy of 92 eV. The spectra of the individual components of the ESCAP resist were obtained by merging the atomic contributions of each molecular species. The spectra for ESCAP and its components are the average over eight structure models.

The theoretical IR spectra were computed on the gas phase molecules optimized with Turbomole 7.2 ^59^ with DFT using the PBE functional^50^ and a Gaussian basis set of triple-ζ valence quality (def2-TZVP).^60–62^ The vibrational frequencies and intensities were calculated at the same level of theory within the harmonic approximation of the optimized geometries. The frequencies were scaled by a factor 0.9923,^63^ and a Lorentzian broadening of 10 cm^–1^ was applied.

## 4 Results and Discussion

In this section, we report the photoelectron spectrum of the ESCAP photoresist upon exposure to photons of 92 eV (13.5 nm) energy. The EUV exposure-driven changes in the valence region were captured in the photoelectron spectra and quantified in terms of peak area changes as a function of the exposure dose. Furthermore, XPS and EUV-induced outgassing mass spectrometry measurements are presented to support the evidence of PAG^–^ decomposition as a result of EUV exposure. Additionally, the FTIR spectra of the ex situ exposed samples are presented to validate the exposure mechanism and to capture the decomposition of PAG^+^ and the co-polymer protecting group.

### 4.1 EUV Photoelectron Spectra

The ESCAP photoresist is composed of five main components: a co-polymer (PHS and PTBMA), a PAG ion pair, and an acid quencher (Figure 1). Thus, the valence region in the energy range of ca. 0–15 eV binding energy arises from ionization out of the molecular orbitals of these building blocks, which also overlap energetically. This makes an unambiguous assignment of peaks in the EUV photoelectron spectrum challenging. To identify the contribution from different components in the valence region of the ESCAP, the photoelectron spectra of the individual components such as co-polymer (mixture of both homo-polymers), a pure PTBMA homo-polymer, a pure PHS homo-polymer, and pure PAG film were measured at 92 eV. In Figure 2a,b, we present the experimental and computed photoelectron spectra of PHS and PTBMA along with the atomic orbital contributions, calculated with density functional theory as described above (Section 3). As illustrated in Figure 2a, the simulated photoelectron spectrum for PHS closely matches that of the measured photoelectron spectrum. Additionally, the separation of computed atomic orbitals contribution from different elements aids the peak identification in the measured spectrum. The molecular orbitals in region ca. 2–12 eV can be assigned to the π bonds on the aromatic ring which are hybridizations of the C­(2p) orbitals and partly to O­(2p) orbitals from the hydroxyl group. The region around binding energy ca. 12–16 eV originates from the C­(2p) bonding orbitals of the C–H, C–C bonds on the chain with added contribution of O­(2p) orbitals. The peaks around ∼20 eV are assigned to the C­(2s) orbitals on the main chain and the aromatic ring, and the peak at ∼27 eV corresponds mainly to the O­(2s) orbitals on the hydroxyl group. Similarly, for PTBMA, in Figure 2b, the simulated photoelectron spectrum matches well with the measured spectrum, and from the atomic orbital contributions, the peaks can be assigned as follows: peaks in the region of ca. 2–10 eV correspond to C­(2p) bonding orbitals of the *tert*-butyl group as well as the main chain with added contributions from C–O–C bonding orbitals on the ester group. The shoulder peak at ∼12 eV involves contribution from the C=O bonding orbitals of the ester group. The peak around ∼20 eV corresponds to the C­(2s) orbitals from the main chain and protection group, and the peak at ∼27 eV originates from the O­(2s) orbitals from the ester group.

**Figure 2:**
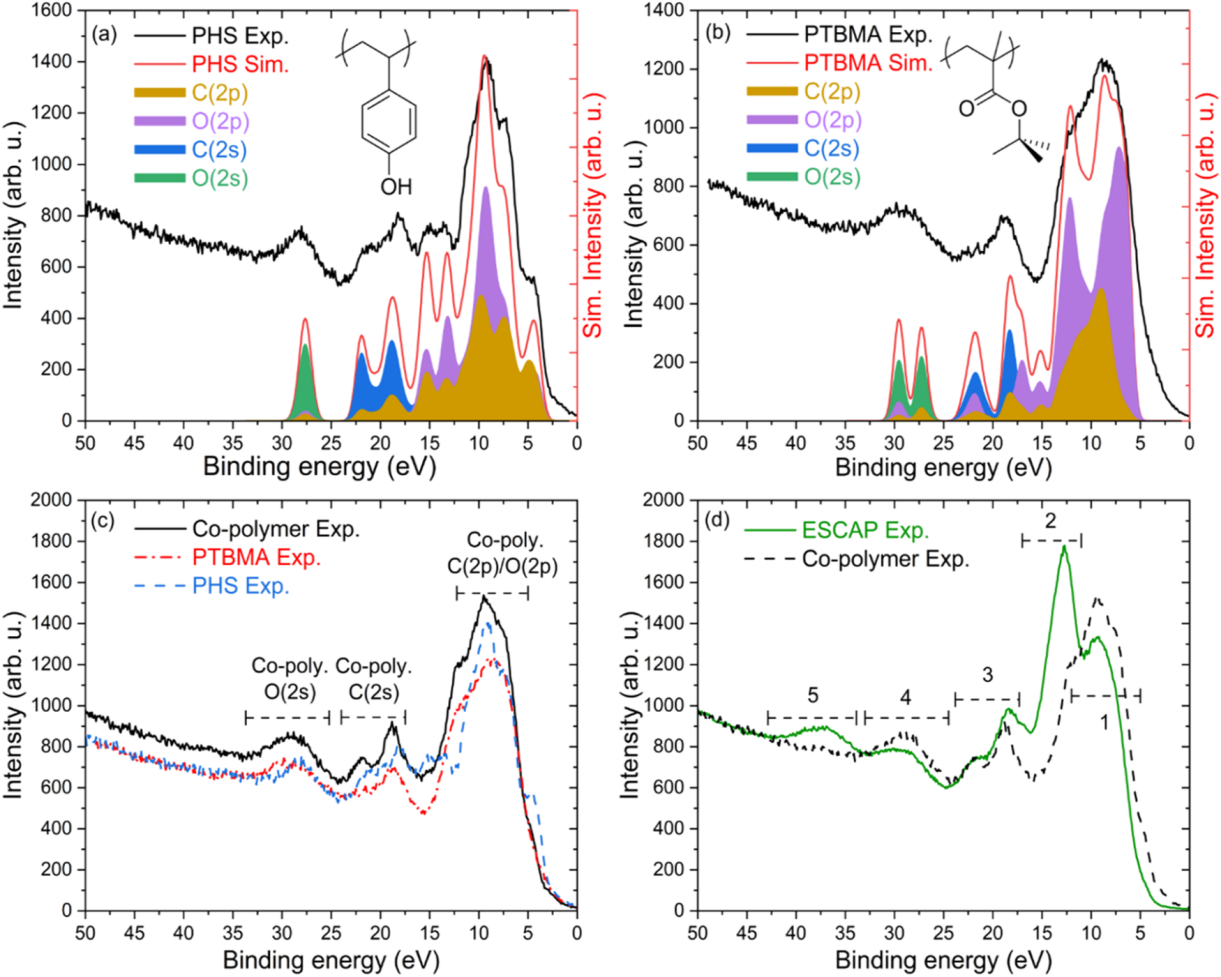
Peak assignment of ESCAP photoelectron spectrum measured at 92 eV photon energy. (a) Photoelectron spectra of pure PHS polymer and computed spectrum of PHS polymer along with highlighted contributions from individual atomic orbitals. (b) Photoelectron spectra of pure PTBMA polymer and simulated spectrum of PTBMA polymer with highlighted contribution from individual atomic orbitals. (c) Photoelectron spectra of co-polymer (approximately ∼52% PTBMA and ∼48% PHS) along with homo-polymers spectra. (d) Photoelectron spectra comparison of ESCAP photoresist and co-polymer. Different regions are allocated to the photoresist components.

Figure 2c presents the photoelectron spectrum of the co-polymer (∼52% PTBMA and 48% PHS), alongside the spectra of the homo-polymers, PHS and PTBMA. By comparing the valence region of PHS and PTBMA with the co-polymer, it is possible to assign the co-polymer peaks to specific bonding orbitals. The peak at ∼27 eV binding energy is mainly attributed to O­(2s), as oxygen is present in both the constituents. In a similar fashion, the region around ∼20 eV arises from C­(2s) atomic orbital on the co-polymer. The region around ca. 2–12 eV arises partly from O­(2p) bonding orbitals from the hydroxyl and ester groups and partly from contributions from π bonds on the ring (PHS), and the C­(2p) orbitals of the *tert*-butyl group (PTBMA). Figure 2d shows a comparison of the measured photoelectron spectrum of the ESCAP photoresist with the co-polymer spectrum. The comparative analysis of these two spectra indicates that distinct energy regions within the photoelectron spectrum of the ESCAP photoresist can be attributed to its individual components. The binding energy regions identified as 1, 3, and 4 predominantly originate from the co-polymer. Additionally, two additional peaks were detected in the ESCAP photoelectron spectrum, at ∼13 eV (region 2) and ∼37 eV (region 5) binding energy, which were absent in the co-polymer spectrum. These peaks may be attributed to other components within the photoresist such as PAG or quencher.

Figure 3a presents the measured photoelectron spectrum of the pure PAG film (nonaflate; PAG^–^ and triphenyl sulfonium; PAG^+^) alongside the spectrum of the ESCAP photoresist. The comparison indicates that the two emerging peaks in regions 2 and 5 could be attributed to the PAG. As previously noted, the PAG comprises an ion pair; thus, it is plausible that one or both components contribute to the emergence of these two new peaks. Consequently, we computed the binding energy and photoelectron spectra for the individual PAG components to facilitate comparison with the photoelectron spectrum of the ESCAP photoresist. The simulated spectrum of the PAG component shows that the PAG^–^ contributes heavily to the two emerging peaks as shown in Figure 3a. Figure 3b presents the experimental and simulated photoelectron spectra of ESCAP, highlighting the contributions from fluorine atomic orbitals in the simulated PAG^–^ spectrum. The comparison reveals that the peak at ∼13 eV (region 2) corresponds to C–F bonding orbitals with a significant F­(2p) coefficient. Additionally, the peak at ∼37 eV (region 5) originates from the F­(2s) atomic orbitals (a similar F­(2s) peak assignment had been done by Brainard et al.^64^). In the case of PAG^+^, the main contribution in the valence region comes from the π bonds on the aromatic rings. Similarly, for the quencher, the valence region is composed mainly of C­(2p) C–C bonding orbitals and C­(2s) atomic orbitals. The computed spectra for PAG^+^ and quencher are shown in Supporting Figure S3. These data, taken together, indicate that PAG^+^ and quencher do not significantly contribute to the two new peaks in the binding energy regions 2 and 5. Therefore, these two new peaks in the photoelectron spectrum of ESCAP are largely associated with the PFAS component (PAG^–^) of PAG.

**Figure 3:**
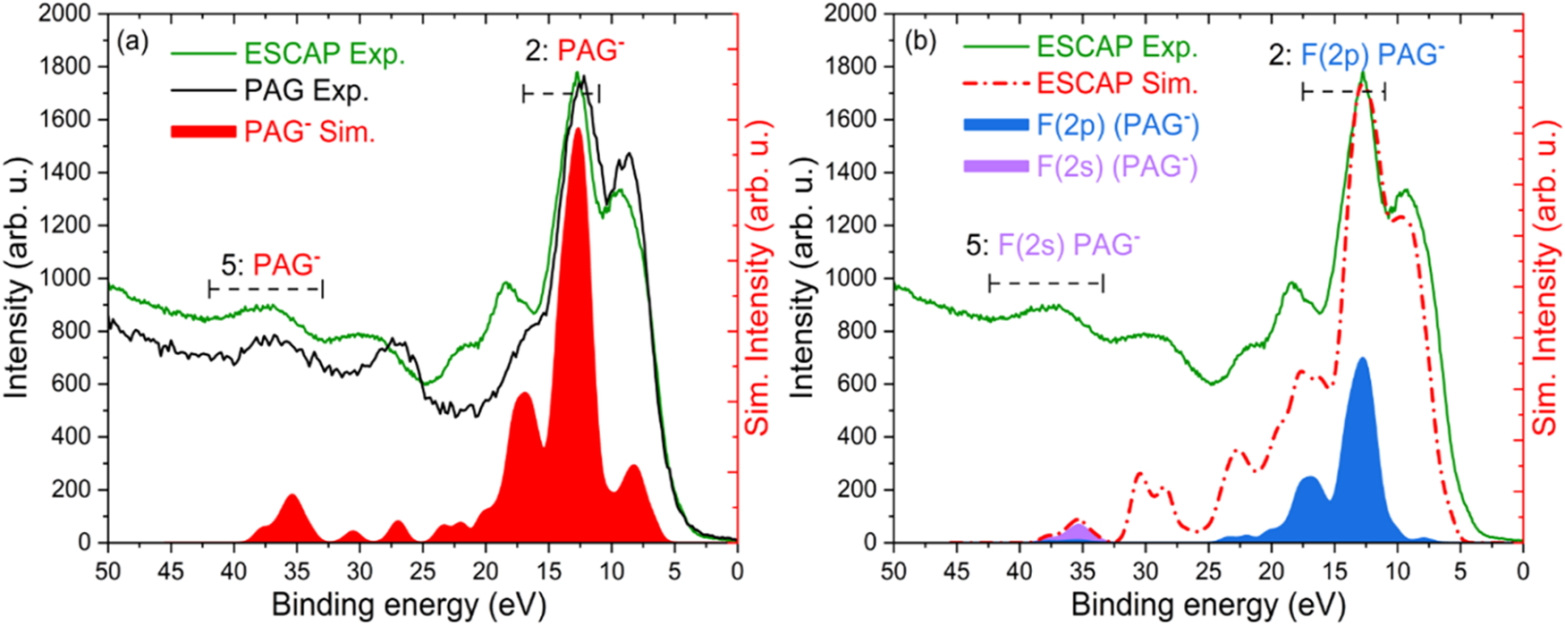
(a) Comparison of photoelectron spectra of pure PAG and ESCAP photoresist along with the computed spectrum of PAG*^–^* component. (b) Comparison of simulated vs measured spectra for ESCAP photoresist with the highlighted contributions of F­(2p) and F­(2s) atomic orbitals of the PAG*^–^* component in regions 2 and 5.

The notable intensity of these new peaks, especially at a relatively low PAG concentration, is attributed to the higher photoionization cross section of fluorine atomic orbitals at 92 eV compared to other elements present in the resist matrix.^58^ The photoionization cross section of fluorine atoms (2s: 0.65 Mb/atom; 2p: 2.57 Mb/atom, total: 3.22 Mb/atom) is five times larger than that of carbon atoms (2s: 0.43 Mb/atom; 2p: 0.21 Mb/atom, total: 0.64 Mb/atom) at a photon energy of 92 eV.^58^ The peak assignment of the ESCAP EUV photoelectron spectrum, particularly the significant presence of PAG^–^ peaks, can be leveraged to obtain chemical sensitivity and to elucidate the decomposition pathways upon EUV exposure. More fundamentally, the presence of photoelectron peaks originating from ionization of the PAG^–^, indicates that during the EUV exposure mechanism, part of the PAG^–^ may participate as *a neutral molecule*, rather than typical conjugate base of the superacid, as proposed in the more widely accepted exposure mechanism. The charge neutrality of the ionized PAG^–^ could have significant consequences on the p*K*_a_ of the superacid, thus altering the efficiency of forming the superacid necessary for the acid-catalyzed deprotection reaction. Such effects could alter the EUV sensitivity of the photoresist and should be considered in reaction schemes of the CAR EUV photoresist. Finally, we note that the EUV PES technique exhibits a distinct sensitivity to fluorine-rich thin films, as well as other elements with high EUV absorption, such as halogen atoms or metals like tin (Sn), zinc (Zn), etc., which are utilized in metal–organic photoresists.^24,65^ This characteristic allows for the tracking of the EUV exposure dynamics in PFAS-based, PFAS-free (halogen-containing), or even metal-based^41^ EUV lithographic materials.

### 4.2 ESCAP EUV Exposure Dynamics

The valence region of the photoelectron spectrum is largely determined by the chemical bonding character of the material. Thus, the EUV-induced chemical changes are expected to manifest the most strongly in this region. Tracking this photoelectron energy range as a function of the exposure dose thus gives the capability to monitor chemical modifications resulting from EUV exposure.

To capture the impact of EUV-induced chemistry in the valence region of the spectrum, we conducted PES measurements in a dynamic manner. This approach involved an extended duration of in situ measurement, resulting in a total accumulated EUV exposure dose on the film of approximately ∼212 mJ/cm^2^, as detailed in Section 2. Figure 4a presents all of the in situ photoelectron spectra (dose: ∼1 mJ/cm^2^ extra per spectrum) displayed as a 3D plot. At first glance, changes in the peak intensity were observed as a function of EUV exposure dose. Figure 4b shows the peak intensity (area under the curve) through dose for the first two peaks in regions 1 and 2. The peak fitting is executed as shown in Supporting Figure S2. As per the peak assignment done in the previous section, these two peaks for the largest part are associated with the co-polymer and PAG^–^ components, respectively. PAG^–^ is the precursor of the superacid that catalyzes the cleavage of the *tert*-butyl protecting group (deprotection) of the ESCAP photoresist and thus is responsible for the main chemical pathway that causes a solubility switch and enables lithographic printing.

**Figure 4:**
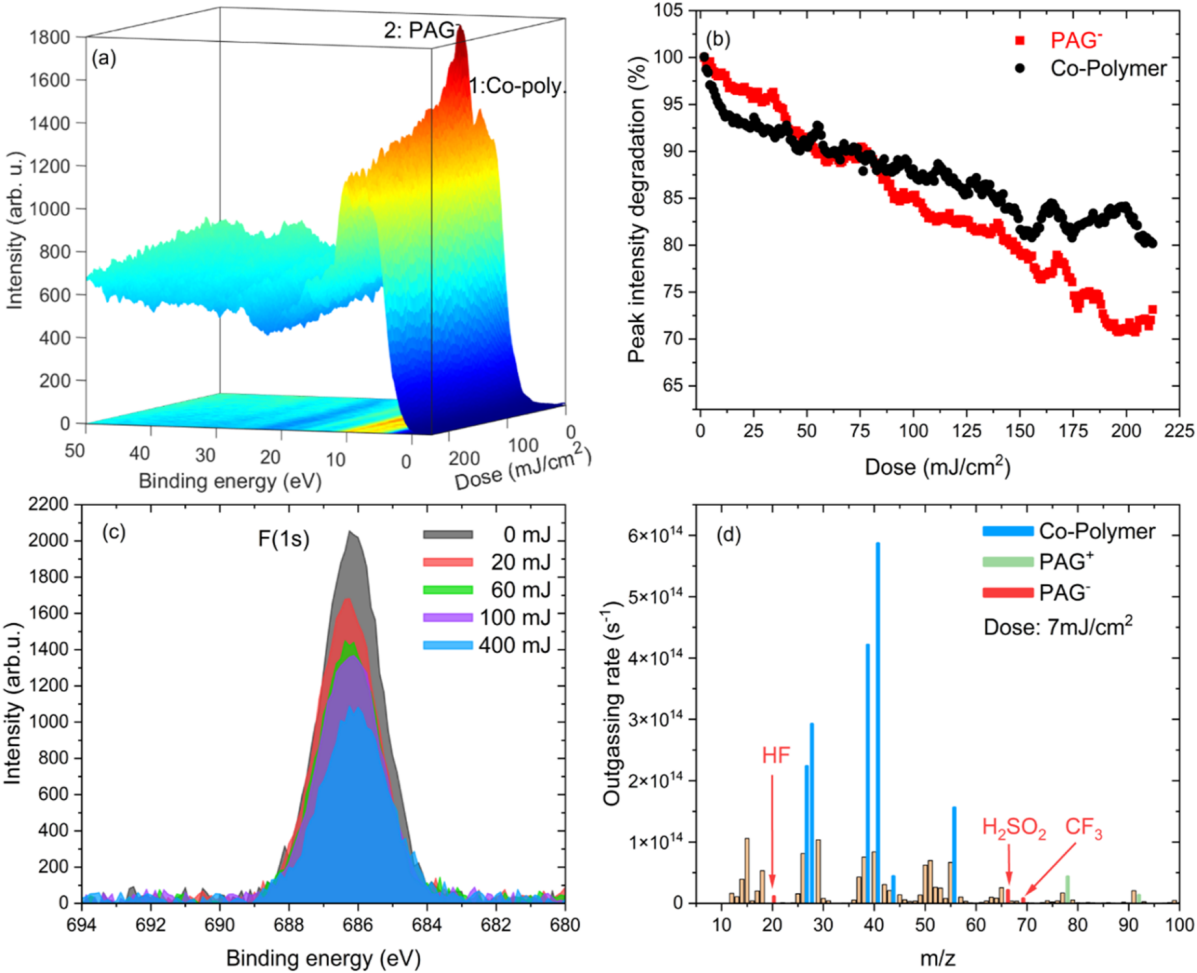
In situ exposure dynamics of ESCAP photoresist films at 92 eV photon energy. (a) Waterfall plot of measured photoelectron spectra of ESCAP as a function of EUV exposure dose of ∼1 mJ/cm*^2^*/scan up to total dose of ∼212 mJ/cm*^2^*. (b) Intensity (area under the curve) of peak 1 and peak 2 plotted as a function of EUV exposure dose. The intensities were extracted as per the procedure explained in Supporting Section S1. (c) F­(1s) peak, XPS of ex situ EUV exposed samples, and (d) EUV-induced mass spectrum of ESCAP photoresist at a low EUV exposure dose of 7 mJ/cm*^2^*.

In Figure 4b, the first peak in region 1, which originates from the co-polymer, shows rapid intensity degradation at lower dose, followed by a gradual reduction. The reduction in intensity associated with the orbitals that have significant carbon and oxygen coefficients might be attributed to the deprotection due to direct photoionization of the co-polymer. Additionally, this could also have contributions from the deprotection triggered by PAG activation during exposure within the resist matrix. This leads to *tert*-butyl and ester group scission in the form of outgassing byproducts such as isobutene and CO_2_ ^12,14,66^ (even at doses as low as ∼10 mJ/cm^2^). The co-polymer decomposition was further observed in the EUV-induced outgassing and FTIR measurements (Section 4.3). Figure 4d presents the mass spectrum data of the ESCAP photoresist at a low EUV exposure dose of ∼7 mJ/cm^2^. The mass spectrum resulting from EUV-induced desorption presents a rather complex spectrum containing signals corresponding to molecular fragments from the co-polymer and PAG ion pair. Previous investigations have thoroughly analyzed the signals arising from the co-polymer and PAG^+^ species.^12,14^ The most prominent signals at *m*/*z* = 56, 41, 39, and 27 can be attributed to isobutene (and its molecular fragments) resulting from cleavage of the *tert*-butyl protecting group of the PTBMA monomer, while signals at *m*/*z* = 28 and 44 correspond to CO and CO_2_ respectively, which occurs during direct cleavage of *tert*-butyl methacrylate moiety^14^ (we note that the signal at *m*/*z* = 28 also contains a contribution from N_2_ but background spectrum recorded on a blank wafer under the same vacuum condition was carefully subtracted).

The second peak in region 2, largely associated with PAG^–^, initially exhibited a gradual reduction in intensity up to ∼30 mJ/cm^2^, whereas at a slightly higher dose (>75 mJ/cm^2^), a faster degradation compared to the co-polymer peak was observed. In the case of PAG^–^, the C–F bonding orbitals (F­(2p) orbitals, see Figure 3b) were found to be the main contributor in region 2. As indicated earlier, the higher photoionization cross section of fluorine atoms results in a prominent fluorine peak in the valence region. Therefore, the degradation of C–F bonding orbitals has a significant effect on the photoelectron spectrum. This suggests a *definitive breakdown of PAG* ^*–*^, especially C–F bonding orbitals under EUV exposure within the ESCAP photoresist. This finding challenges the previous assumption in the EUV exposure mechanism for CAR, which assumes that PAG^–^ remained *chemically intact* as a precursor to the superacid during EUV exposure.^13,16,19,21,29,67^ One potential pathway for PAG^–^ decomposition involves defluorination. Morikawa et al.^68^ observed a similar decomposition for polyvinyl fluoride, a fluorine-based photoresist. The authors noted that the degradation of the fluorine photoelectron peak was dependent on the dose (UV exposure) and was correlated with HF outgassing.

The PAG^–^ decomposition due to EUV exposure was further supported by XPS and EUV-induced outgassing measurements. Figure 4c presents the XPS measurements on ex situ exposed ESCAP photoresist. The data indicated a reduction in the intensity of the F­(1s) peak correlated with increased EUV exposure dose. This suggests a potential breakdown of PAG^–^ through defluorination, even at lower exposure doses. Furthermore, Figure 4d presents the mass spectrum data highlighting the signals originating from PAG decomposition. While much of the mass spectrum is dominated by signals from the co-polymer (which is present at nearly ×10 the concentration of the PAG^+^ and PAG^–^, c.f. Figure 1), distinct signals from the PAG ionic pair are observed. Signals at higher *m*/*z* ratios, near 78 and 92, can be attributed to the release of benzene and toluene, which corresponds to fragmentation of the PAG^+^.^12^ However, a group of peaks near *m*/*z* = 69, 66, and an isolated signal at *m*/*z* = 20 have, to our knowledge, not yet been identified in the literature. The uniqueness of a mass at *m*/*z* = 20 is most likely due to HF outgassing from the PAG^–^ as a result of C–F bond cleavage upon EUV exposure. Additionally, a cluster of peaks near *m*/*z* = 66 is representative of H_2_SO_2_ and could be the result of C–S bond cleavage, thus separating the sulfate group from the perfluoroalkyl backbone of the PAG^–^. Finally, we observe a signal at *m*/*z* = 69, which corresponds to CF_3_ fragments that come from the CF_3_ moiety of the C–F chain of the PAG^–^. We note that these unique signals of the PAG^–^ are not observed in the mass spectrum of the co-polymer-only sample (see Supporting Figure S4), thus further confirming that the signals result from the PAG^–^ decomposition. While at this time the complex nature of ionization, fragmentation, and interaction of excited PAG^–^ molecules prevents a full description of the mechanism for PAG^–^ degradation, we can assume from the above data that cleavage of C–F and C–S bonds is a possible reaction pathways. Furthermore, the presence of HF in the outgasses spectrum indicates that F anions or radicals are being generated, which can then abstract a proton from the resist matrix. In order to elucidate the exact mechanism, detailed studies are needed on the isolated PAG^–^ system, and these studies are currently planned for future work.

These results show that despite the high EUV dose applied for the in situ PES measurements (>200 mJ cm^–2^), the breakdown of the PAG^–^ occurs at lithographically relevant doses. This suggests that PAG^–^ decomposition occurs alongside the reaction necessary for image formation mechanism. The above data and reasoning indicate that decomposition of PAG^–^ could have large implications for the lithographic printing capabilities. For instance, the absence of PAG^–^ could result in an increased number of stochastic defects (i.e., regions of unactivated photoresist) as locally fragmented PAG^–^ molecules would not be able to participate in the acid-catalyzed solubility switch. Furthermore, the sensitivity of the resist could be reduced, especially if the relative concentration of PAG^–^ molecules (referred to as *PAG loading* in lithography literature) is linked to the required dose-to-size (which is often the case). Additionally, reducing the active PAG^–^ concentration could result in local variations of the acid-quencher balance, which might influence the line-edge and line-width roughness of nanoscale features. However, we note that dedicated lithographic studies with various PAG loadings and structures would be needed to verify these effects, which are beyond the current scope of the paper. Finally, the breakdown of the PAG^–^, itself a PFAS molecule, could result in new fluorinated byproducts that leave the resist film through outgassing (as suggested by the EUV-induced outgassing mass spectrum data).

### 4.3 Fourier Transform IR Spectroscopy of Ex Situ Exposed ESCAP

The EUV PES results, combined with theoretical modeling and peak assignment, allow for the tracking of in situ EUV exposure dynamics. However, the presence of overlapping valence levels of the many constituents of the ESCAP resist presents a significant challenge for isolating chemical dynamics from all of the components of the photoresist. To further enhance and support the findings from the EUV PES data, we performed high-sensitivity FTIR measurements on ex situ exposed ESCAP samples. In Figure 5, we show the FTIR spectra of the ESCAP photoresist at exposure doses of 0, 40, and 200 mJ/cm^2^ (for additional data and exposure doses, see Supporting Figure S5). Regions of the FTIR spectra that exhibit a change through exposure dose are marked with either an asterisk (*), corresponding to chemical changes in the PTBMA component of co-polymer, or a cross (+), which corresponds to chemical dynamics of the PAG^+^ (see Supporting Figure S6 for additional simulated IR spectrum from individual components). We note that peaks associated with the PHS component do not significantly change through exposure dose, nor do we observe any peaks that can be conclusively linked to the quencher or PAG^–^ species.

**Figure 5:**
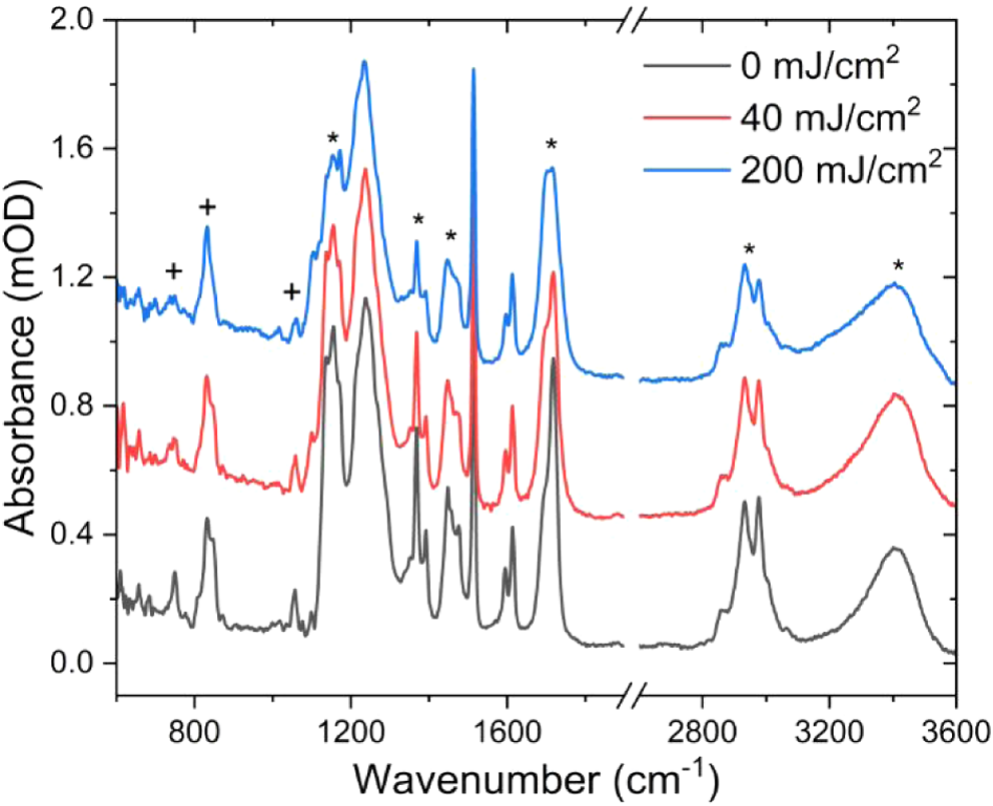
High-sensitivity FTIR spectra of ex situ exposed ESCAP films at exposure doses of 0, 40, and 200 mJ/cm*^2^*. The asterisks (*) highlight peaks in the FTIR spectrum corresponding to changes in the PTBMA polymer, which contains the cleavable protecting group of the ESCAP system. The plus (+) symbols highlight peaks corresponding to vibrational modes of the PAG^+^ component. The spectra are unaltered, aside from a baseline offset for clarity. The region from ∼1900 cm*^–^* ^1^ to 2700 cm^–1^ does not contain any vibrational modes and is cropped for clarity (see Supporting Figure 5 for full FTIR spectra).

The observed changes in the FTIR spectra are a direct result of the same EUV-induced exposure dynamics that occur within the ESCAP system during the in situ and ex situ exposures. Focusing on peaks associated with the PTBMA component of the co-polymer (* symbols), we observe the following trends. First, the broad peaks in the region of 1100–1200 cm^–1^, corresponding to O=C–O–C vibrations of the ester moiety of the PTBMA polymer unit, reduces and broadens with increasing exposure dose. This suggests cleavage of the O–C ester bond and/or direct cleavage of the *tert*-butyl methacrylate moiety. The doublet observed at ∼1350 cm^–1^ results from CH_3_ bending vibrations of the *tert*-butyl protecting group on the PTBMA unit exhibits further reduction with increased exposure dose (Figure 6a). Additionally, a significant reduction in the intensity of the peaks in the CH stretching region is observed (Figure 6b). This further confirms the loss of the *tert*-butyl group as a function of the exposure dose. Finally, we also observe a significant broadening of the OH stretch in the region of 3200–3600 cm^–1^ (Figure 6b). This is the result of protonation of the ester moiety following *tert*-butyl cleavage, thus resulting in a carboxylic acid, which is the desired product to cause the solubility switch to create positive tone resist behavior when developed in aqueous base solvents. The deduced chemical dynamics of the protecting group from the FTIR measurements are in line with previously reported studies on EUV-induced chemical dynamics in the ESCAP system.^12,16,34^ We also observe a significant reduction of the carbonyl peak near 1710 cm^–1^ (Figure 6c), which is a result of CO_2_ loss upon direct cleavage of the *tert*-butyl methacrylate from the polymer backbone, as also observed in outgassing measurements^12^ (see Supporting Figure S7 for simulated IR spectra of PTBMA, with/without outgassing byproducts). Moreover, these findings directly align with in situ EUV PES results, where we measured the reduction in intensity of peaks originating from co-polymer (see Figure 4).

**Figure 6:**
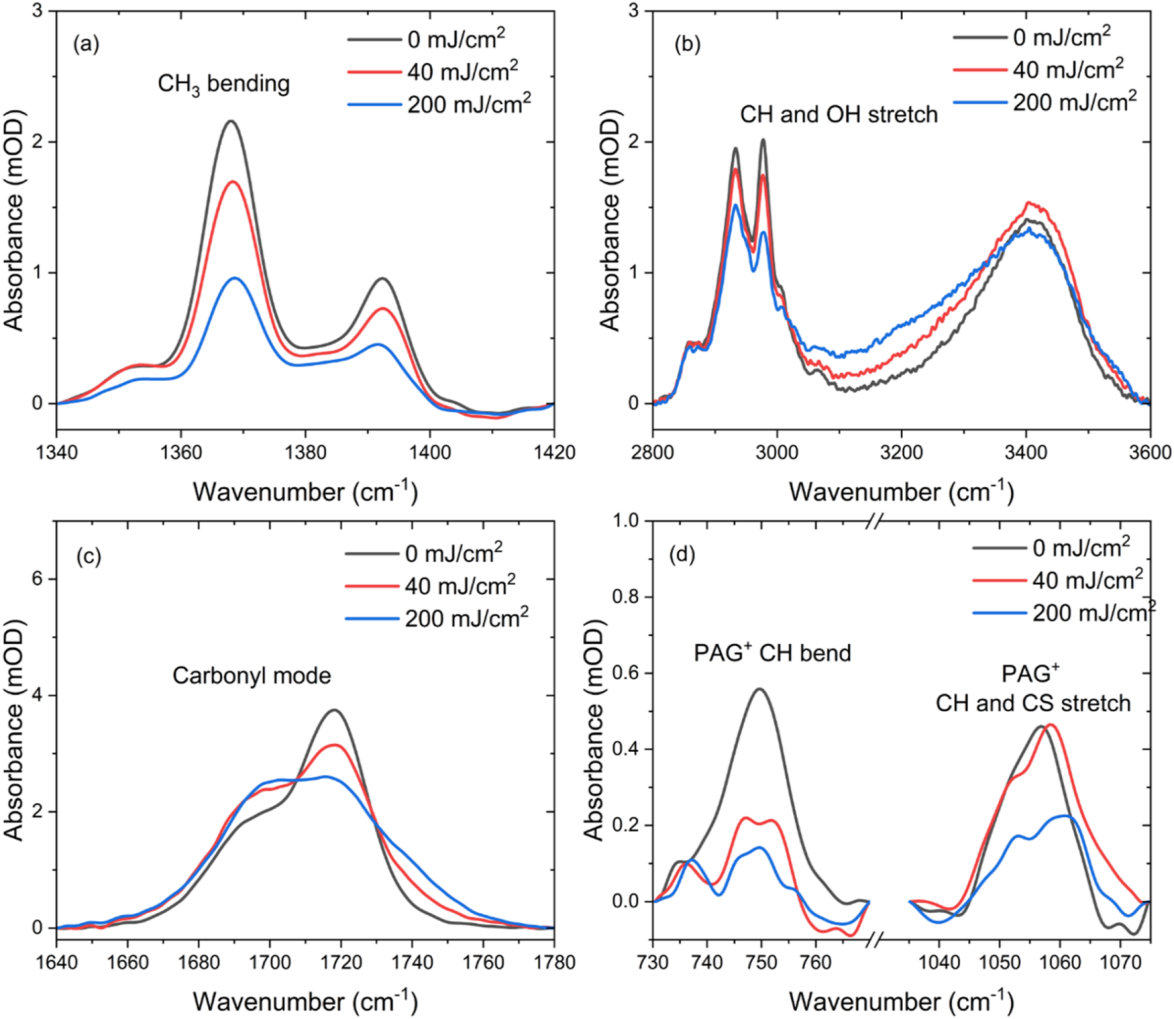
Regions of interest in the FTIR spectra of the ex situ exposed ESCAP photoresist as a function of exposure dose. The regions of interest correspond to (a) CH*_3_* bending of the *tert*-butyl protecting group, (b) CH and OH stretching regions, (c) carbonyl stretching of the BMA moiety, and (d) PAG^+^ CH bending modes and PAG^+^ coordinated CH–CS stretching. Spectra are uncorrected aside from a baseline subtraction within each region of interest.

In addition to following the chemical dynamics of the protecting group, our high-sensitivity FTIR setup also enables us to directly detect vibrational peaks resulting from the PAG^+^ ion (+ symbols in Figure 5). These low-frequency peaks correspond to CH bending modes of the aromatic rings of the PAG^+^ (∼750 cm^–1^) and coordinated CH–CS vibrational modes of (4-methylphenyl) diphenyl sulfonium (∼1050 cm^–1^) (see Supporting Figure S8). The reduction in the intensity of these peaks (Figure 6d) corresponds directly to the breakdown of the PAG^+^ ion, which is believed to be the first step in PAG activation that ultimately results in the subsequent release of the conjugate superacid. Interestingly, we observed a complete reduction of the PAG^+^ peaks at a nominal exposure dose of ∼40 mJ/cm^2^, despite the film thickness remaining constant within this exposure dose range (see Supporting Figure S9). These findings suggest that the PAG^+^ breakdown is mediated via the release of one (or more) of the aromatic rings, which could result in the creation of aromatic free radicals.^69^ Along these lines, we note that free aromatic rings have been observed in outgassing experiments and interpreted as the breakdown/activation of the PAG^+^.^12^ Moreover, recent massive cluster time-of-flight mass spectrometry experiments have also observed polyaromatic PAG^+^ species being formed after exposure^70^ further supporting the observations from the FTIR measurements.

While the FTIR measurements confirm the overall exposure mechanism and provide strong evidence that the observed chemical dynamics in EUV PES are the result of the same exposure mechanism, we note that the FTIR spectrum does not show any signature that can be conclusively related to the PAG^–^ component. This lack of observation is the combination of two coupled effects, a low PAG^–^ percentage in the films compared to the co-polymer, as well as significant overlap of PAG^–^ vibrational peaks with the co-polymer itself (see Supporting Figure S6). However, in our EUV PES measurements, we observed a clear reduction of peak intensity in region 2, which, via simulations, we have shown is the result of PAG^–^ decomposition. This surprising result indicates that PAG^–^ could also experience its own EUV-exposure-induced dynamics, in addition to the desired chemical dynamics occurring at low exposure doses (i.e., release from the PAG^+^ and protonation to form the superacid). This potentially parallel reaction pathway could be a source of cross-linking that is observed at higher exposure doses in the ESCAP system^12,34^ as well as a source of stochastic defects as fragmented PAG^–^ would likely have a reduced effectiveness in acid-catalyzed deprotection. For this system, the lack of signature in FTIR measurements for PAG^–^ further highlights the power of a multitechnique characterization approach involving in situ EUV PES combined with advanced theory for unraveling complex chemical exposure dynamics in EUV photoresists.

## 5 Conclusions

In this work, we present a comprehensive experimental characterization of the ESCAP photoresist. The performance of EUV photoresists is intrinsically linked to chemical processes induced by exposure, such as electron emission, molecular fragmentation, and electron attachment within the resist film upon EUV photon absorption. To elucidate the exposure mechanism, the model photoresist was examined by using table-top EUV photoemission and FTIR spectroscopy techniques.

Through the measurement of the photoelectron spectra of homopolymers (PHS, PTBMA) of the individual polymer components, the full resist co-polymer, the PAG, and the complete ESCAP formulation, combined with computed photoelectron spectra using density functional theory, we identified the contributions from various resist components in the valence region of the ESCAP photoelectron spectrum. A notable contribution, at ∼13 eV binding energy, has been distinctly identified to be attributed to the PAG^–^. Its strong impact is caused by the substantial EUV absorption cross section of the fluorine atoms.

The evolution of the EUV exposure process was captured through long EUV exposure with simultaneous in situ EUV PES measurements. These measurements offered substantial insights into the interaction between EUV radiation and the components of the full resist matrix. For the first time, the degradation of peaks associated with the PAG^–^ component (PFAS molecule) due to EUV exposure has been observed for the CAR photoresist. This observation challenges the previously held assumption that PAG^–^ remains chemically intact during exposure. As a result, it implies that the ionization of PAG^–^ needs to be considered in a full understanding of the exposure mechanism of CAR photoresist systems.

Additionally, FTIR measurements provided complementary information on the exposure-driven degradation of the PTBMA units in the co-polymer (as also observed in EUV PES spectra) and the PAG^+^ components, revealing the degradation pathways leading to CO_2_ outgassing and direct protection group scission upon EUV exposure. Thus, it is imperative to consider these recent findings on the EUV exposure-induced degradation of PFAS components in photoresist for the design and improvement of next-generation EUV photoresist systems. Finally, a thorough investigation of the breakdown of PFAS vs PFAS-free (and even fluorine-free) PAG compounds should be explored in order to evaluate their stability during EUV exposure, which could also help to drive further developments in PFAS-free chemistry of EUV photoresists. Additionally, metal–organic photoresists containing high-EUV-absorbing elements such as tin (Sn) and zinc (Zn) present promising candidates for investigation using the EUV PES technique, owing to the distinct Sn­(4d) and Zn­(3d) features observed within the valence band region. Such studies will be the subject of future work.

## Supplementary Material



## Notes

EUV photoelectron spectrum peak fitting procedure, computed photoelectron spectrum for PAG^+^ and quencher, EUV-induced outgassing of co-polymer, computed FTIR spectra, thickness measurements, and thermal effects during EUV exposure (PDF)
