# Supplementary material for: Resolving In Situ Exposure Dynamics in a Chemically Amplified EUV Photoresist Using Table-Top EUV Photoemission Spectroscopy
Source: ACS Appl Mater Interfaces. 2025 Sep 5;17(37):52567–79. doi: 10.1021/acsami.5c09589 (PMC12447401; doi:10.1021/acsami.5c09589)
Supplement: Supplementary file 1 [file am5c09589_si_001.pdf]

Supporting Information

RESOLVING IN SITU EXPOSURE DYNAMICS IN A CHEMICALLY AMPLIFIED EUV PHOTORESIST USING TABLE-TOP EUV PHOTOEMISSION SPECTROSCOPY

Dhirendra P. Singh[1*], Laura Galleni[1,2], Faegheh S. Sajjadian[1,2], Ivan Pollentier[1], Fabian Holzmeier[1], Geoffrey Pourtois[1], Stefan De Gendt[1,2], Michiel J. van Setten[1], Thierry Conard[1], John S. Petersen[1], Paul A. W. van der Heide[1], and Kevin M. Dorney[1]

[1]Imec, Kapeldreef 75, 3001 Leuven, Belgium

[2]Department of Chemistry, KU Leuven, Celestijnenlaan 200F, 3001 Leuven, Belgium

*Correspondent email: Dhirendra.Pratap.Singh@imec.be

Phone: +32 477332491

S1. Processing and peak deconvolution of the in-situ EUV photoemission spectra

The recorded, in-situ EUV photoemission spectra were further processed for quantitative analysis using a two-step procedure. First, a Tougaard baseline was applied to the raw spectra between the ranges of 0- 60 eV binding energy (supplementary Figure S1). This polynomial baseline was then subtracted from the measured photoemission spectra to remove the

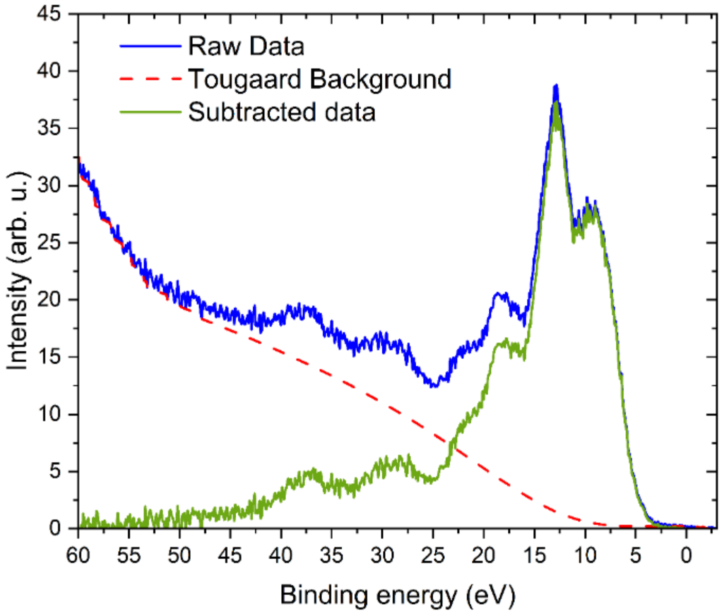

Figure S1. Exemplary EUV photoemission spectrum of the ESCAP photoresist, showing the background subtraction process. The raw EUV photoemission spectrum (blue) is truncated to binding energies >60 eV and a Tougaard background is calculated over the energy range of 0-60 eV. The calculated background corresponding to contributions of secondary electrons (red) is then subtracted from the raw spectrum to yield a background-subtracted spectrum (green) for fitting analysis.

contribution of secondary electrons, which are not included in the theoretical modelling. Next, a peak deconvolution routine based on non-linear least-squares minimization was implemented using CasaXPS software. For peak deconvolution, a basis set composed of mixed Gaussian-Lorentzian peaks (90/10) was used to account for effects of various broadening mechanisms in the recorded data. In our experiment, the predominant sources of broadening are due to a) the

bandwidth of the table-top EUV source at 13.5 nm ($\Delta\lambda/\lambda \sim 10^{-2}$) and b) the electrostatic environment inside the polymer-based resist matrix, while lifetime broadening only accounts for a minor portion of the observed experimental broadening. We find that a basis set composed of 7 Gaussian-Lorentzian peaks accurately reproduces the experimental spectra (supplementary Figure S2 for a selected spectrum). For all the spectra we achieved a residual STD of < 1.

The intensity shown in Figure 3b was calculated by extracting the area under the curve based on the fitting depicted in supplementary Figure S2. In Figure 3b, for the co-polymer peak in region 1, the plotted intensity is the sum of the areas of gaussians peak1a and peak1b. For the

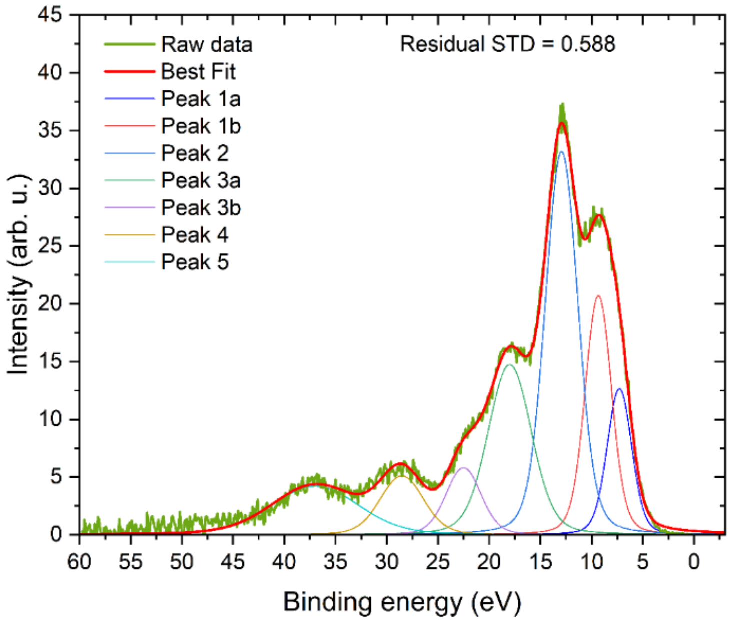

Figure S2. Exemplary background-subtracted (green) and fitted (red) EUV photoelectron spectrum of the ESCAP material in the valence and deep-valance region. The raw data (blue line, top panel) is well described by a basis set of 7 mixed Gaussian-Lorentzian peaks (90/10 ratio).

PAG$^-$ peak in region 2, the plotted peak intensity is the area of gaussian peak 2. The intensities plotted in Figure 3b are a 10-point moving average through dose for both the PAG$^-$ and co-polymer.

S2. Computed photoelectron spectra of PAG$^+$, and Quencher.

The simulated photoelectron spectra for PAG$^+$ and quencher are present in supplementary Figure S3 in comparison with PAG$^-$ and experimental spectrum of full photoresist. The simulated spectra takes into account the photoionization cross-section of all the elements present in the individual component of the photoresist. This comparison shows that the

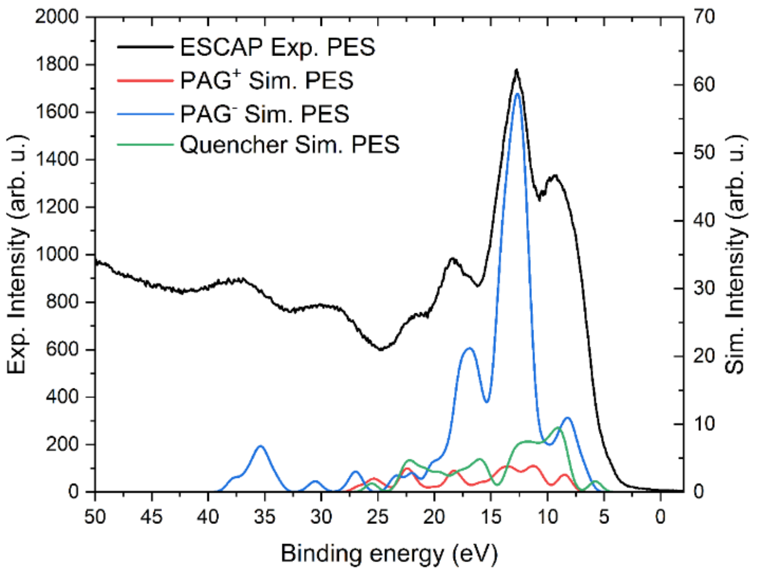

Figure S3. The computed photoelectron spectra of PAG$^+$ and Quencher are shown in comparison with simulated PAG$^-$ and experimental photoelectron spectra of ESCAP photoresist.

contribution of PAG^+ and quencher in the region 2 around 13 eV binding energy is very minimal and only PAG^- is the highest contributor to the peak intensity.

S3. EUV-induced outgassing of co-polymer

The mass spectrum of co-polymer at EUV exposure dose of ~7mJ/cm^2 is presented in supplementary Figure S4. The species at m/z ratio of 20, 66, 69 (for PAG^-) and 78, 79 (for PAG^+) which are assigned to PAG component are not observed in co-polymer mass spectrum.

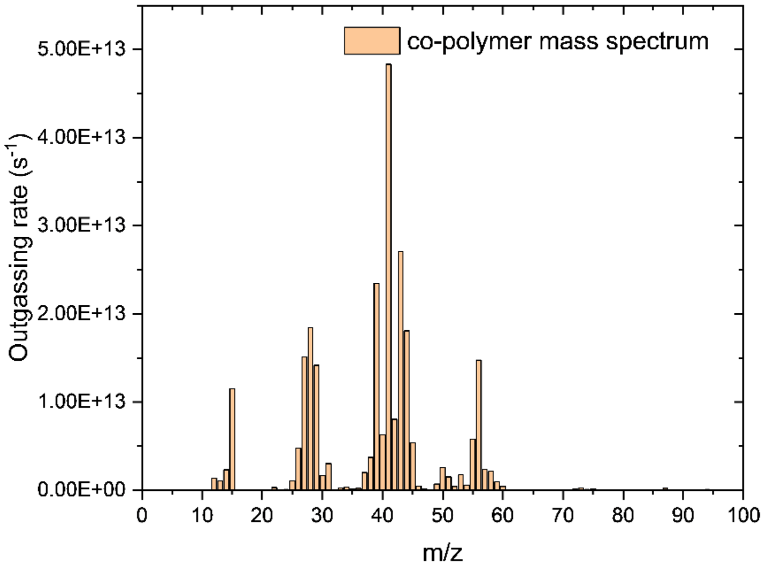

Figure S4. EUV-induced outgassing mass spectrum of co-polymer at dose 7mJ/cm^2.

S4. Full FTIR Spectra of ex-situ exposed ESCAP photoresist and computed FTIR spectra

In this section, we show extended FTIR data on the ex-situ exposed ESCAP photoresist. Figure S5 shows full FTIR spectra as a function of exposure dose, highlighting the fingerprint and

high-frequency CH and OH stretching regions. The spectra are unprocessed aside from a baseline offset for ease of viewing.

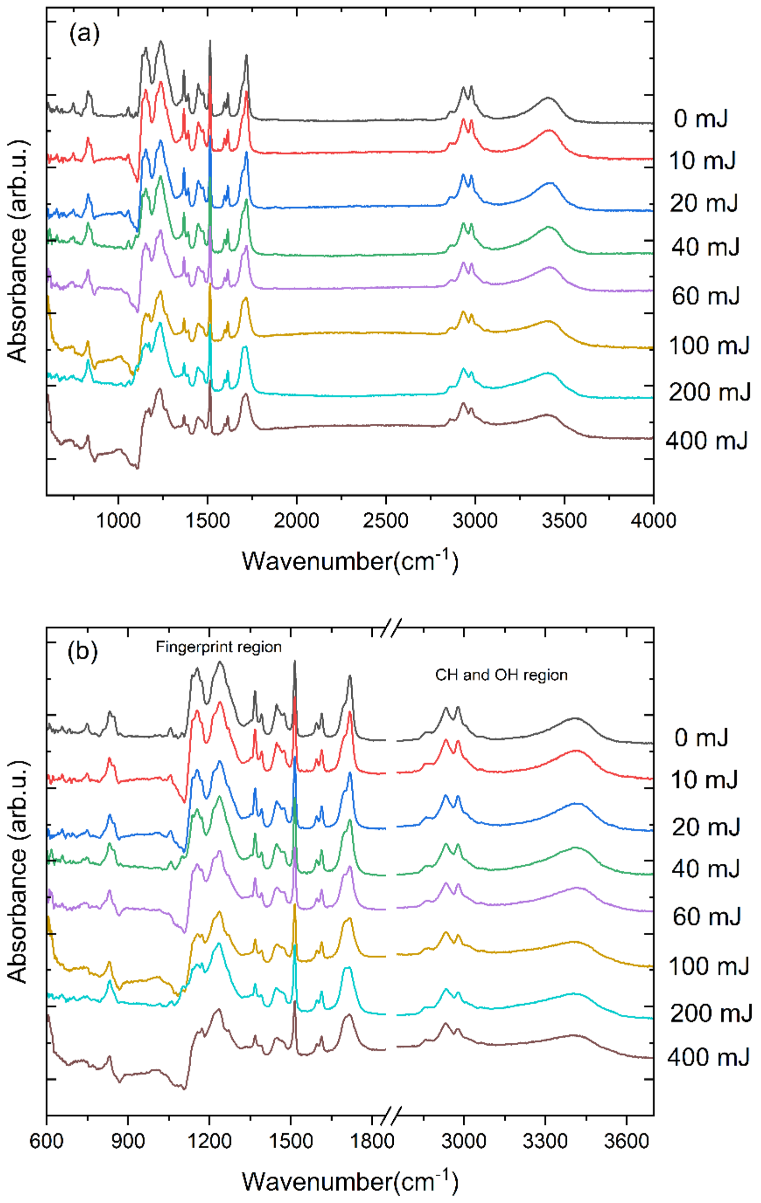

Figure S5. Full FTIR spectra of the ex-situ exposed ESCAP photoresist as a function of exposure dose. (a) Full scale FTIR spectra of the ex-situ exposed ESCAP photoresist. (b) Fingerprint and CH and OH stretching regions of the FTIR spectra in (a).

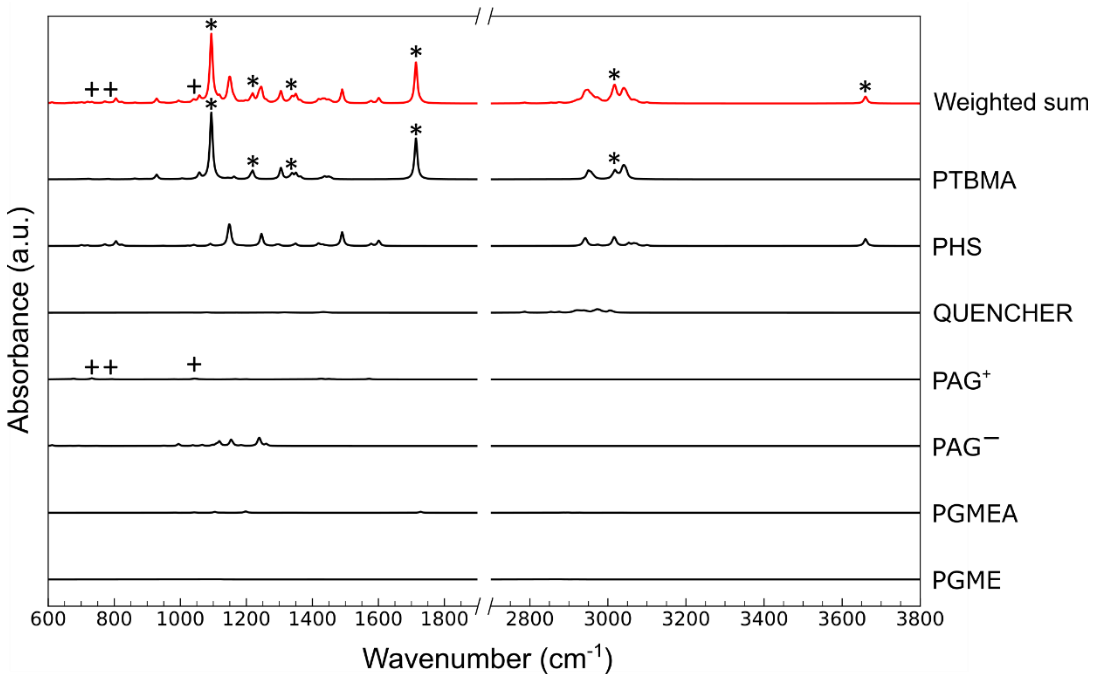

Figure S7. Simulated IR spectra of each separate component of the ESCAP photoresist in the gas phase and their sum weighted by their molar ratio for comparison with Figure 5. All spectra are weighted by their molar ratio. The peaks highlighted by asterisks (*) and plus (+) symbols correspond to the peaks undergoing changes upon exposure (compare with Figure 5 and 6).

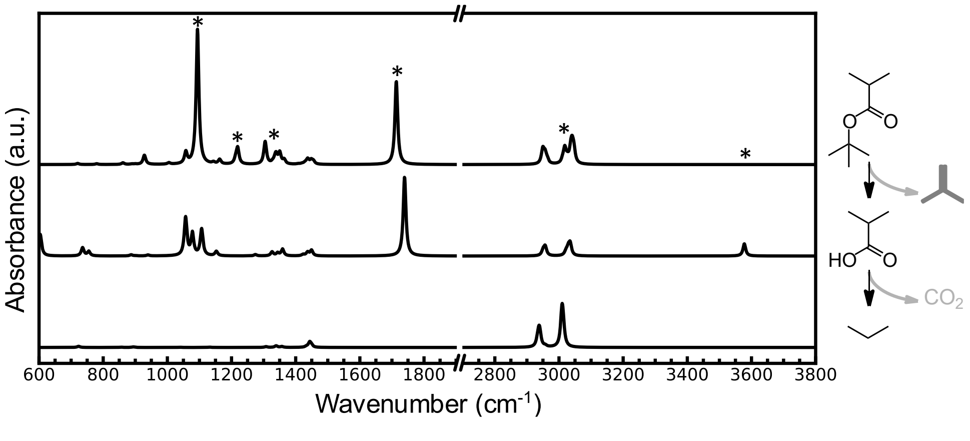

Figure S6. Simulated IR spectra of (top) one hydrogen-terminated repeat unit of PTBMA in the gas phase, (middle) the same molecule after the removal of the tert-butyl and protonation of the deprotected group, resulting in a carboxylic acid, which is the desired product to cause the solubility switch and (bottom) after release of CO_2. The asterisks (*) highlights the regions where the intensity changes upon exposure (compare with Figure 6).

S4.1 Vibrational frequencies of the PAG^+ molecule.

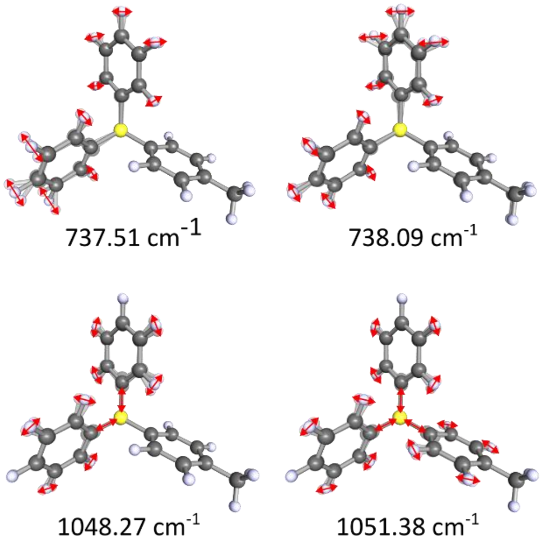

Figure S8. Selected vibrational modes of the PAG^+ molecule computed with density functional theory at the PBE/def2-TZVP level of theory. These coordinated modes are isolated from other components in the resist matrix, and reduction of these modes via increasing EUV exposure indicates breakdown of the PAG^+. Gray=carbon, white=hydrogen, yellow=sulfur.

S5. Measured thickness of ex-situ exposed ESCAP photoresist

Spectroscopic ellipsometry measurements were utilized to measure the thickness loss of the ex-situ exposed ESCAP samples, which occurs to due to shrinkage upon EUV exposure. In Figure S9, we show the resulting mean and standard deviation of the resist film thickness at various exposure doses as measured across the entire resist sample. In general, a gradual reduction of photoresist film thickness is observed as a function of exposure dose, which is a consequence of material loss via outgassing. The measured thickness values were used to scale

the measured FTIR signals to account for effects of film thickness loss during quantitative analysis of the FTIR data.

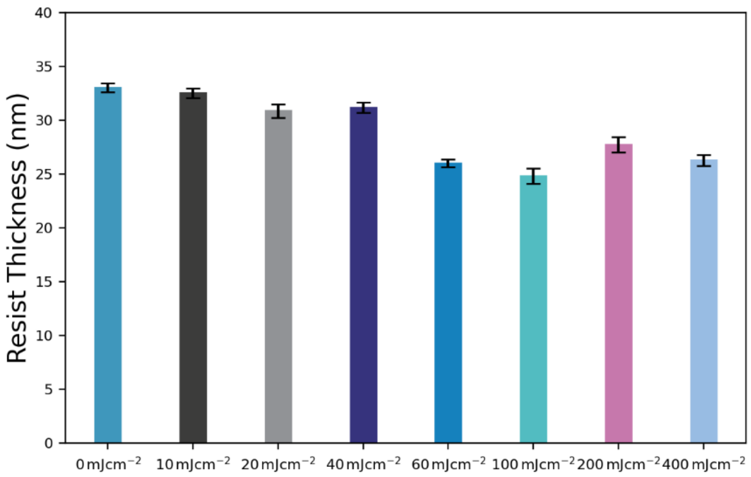

Figure S9. Remaining thickness of the ESCAP photoresist for each ex-situ exposure dose measured via FTIR and/or XPS. The error bars indicate the 1-sigma standard deviation of the measured thickness across the sample.

S6. Calculation of thermal heating of ESCAP resist during EUV exposure

This section provides an analysis of the thermal effects induced by EUV exposure. The change in the temperature of the photoresist film was calculated using Fourier's law,

$$\Delta T = \frac{P.A}{4\pi h r^2} \quad \text{S(1)}$$

where P is EUV power (W), A is EUV absorption, h is the heat transfer coefficient (W/m^{2}K), and r is the radius of the EUV spot (m).

In case of EUV PES experiments, the incident EUV power (P) is ~1 nW, with a beam diameter of 120 x 70 μm^2 (radii, r, of 60 and 35 μm). Given a nominal absorption coefficient (α) of ~5 μm^{-1}, which is typical for CAR species[1], the absorbance (A) of our 30-nm thick ESCAP samples is ~0.15. By employing typical thermal conductivity (k) of ~0.3 W/mK for

photoresists, which yields a heat transfer coefficient (h) of ~10^7 W/m^2K [2]. The calculated temperature change was, $\Delta T = 2x10^{-9}\ K$ for resist film and $\Delta T = 2.2x10^{-3}\ K$ for the silicon substrate.

For EUV induced mass spectroscopy, the results were obtained with a more powerful EUV source with a bigger spot size (3 mW and 2.5 mm radius). Here, the localized heating is calculated to be ΔT = ~2.3×10^{-6} and 2.3 K for the resist and silicon substrate, respectively.

References

(1) Fallica, R.; Haitjema, J.; Wu, L.; Castellanos, S.; Brouwer, F.; Ekinci, Y. Absorption Coefficient and Exposure Kinetics of Photoresists at EUV. In *Extreme Ultraviolet (EUV) Lithography VIII*; 2017; Vol. 10143, p 101430A. https://doi.org/10.1117/12.2257240.

(2) Fallica, R.; De Simone, D.; Hopkins, P.; Jones, A.; Gaskins, J. Thermal Conductivity of Underlayers for EUV Lithography and Its Effect on Sensitivity of Metal Oxide Resist. In *Optical and EUV Nanolithography XXXVIII*; Burkhardt, M., van Lare, C., Eds.; SPIE, 2025; p 60. https://doi.org/10.1117/12.3051713.
